# Comparison of clinical safety and efficacy of dexmedetomidine, remifentanil, and propofol in patients who cannot tolerate non-invasive mechanical ventilation: A prospective, randomized, cohort study

**DOI:** 10.3389/fmed.2022.995799

**Published:** 2022-08-30

**Authors:** Mine Altınkaya Çavuş, Serife Gökbulut Bektaş, Sema Turan

**Affiliations:** ^1^Kayseri City Hospital, Republic of Turkey Ministry of Health Sciences, Kayseri, Turkey; ^2^Ankara City Hospital, Ankara, Turkey

**Keywords:** non-invasive ventilation, chronic obstructive pulmonary disease, dexmedetomidine, remifenthanyl, propofol, intensive care

## Abstract

**Background and objectives:**

Non-invasive ventilation (NIV) is used in intensive care units (ICUs) to treat of respiratory failure. Sedation and analgesia are effective and safe for improving compliance in patients intolerant to NIV. Our study aimed to evaluate the effects of dexmedetomidine, remifentanil, and propofol on the clinical outcomes in NIV intolerant patients.

**Methods:**

This prospective randomized cohort study was conducted in a tertiary ICU, between December 2018 and December 2019. We divided a total of 120 patients into five groups (DEX_L_, DEX_H_, REM_L_, REM_H_, PRO). IBM SPSS Statistics 20 (IBM Corporation, Armonk, New York, USA) was used to conduct the statistical analyses.

**Results:**

The DEX_L_, DEX_H_, REM_L_, and REM_H_ groups consisted of 23 patients each while the PRO group consisted of 28 patients. Seventy-five patients (62.5%) became tolerant of NIV after starting the drugs. The NIV time, IMV time, ICU LOS, hospital LOS, intubation rate, side effects, and mortality were significantly different among the five groups (*P* = 0.05). In the groups that were given dexmedetomidine (DEX_L_, and DEX_H_), NIV failure, mortality, ICU LOS, and hospital LOS were lower than in the other groups.

**Conclusion:**

In this prospective study, we compared the results of three drugs (propofol, dexmedetomidine, and remifentanil) in patients with NIV intolerance. The use of sedation increased NIV success in patients with NIV intolerance. NIV failure, mortality, ICU LOS, IMV time, and hospital LOS were found to be lower with dexmedetomidine.

## Introduction

Non-invasive ventilation (NIV) is frequently used in intensive care units (ICUs) to treat of acute exacerbations of chronic obstructive pulmonary disease (COPD). This supportive treatment reduces both the need for invasive ventilation (IV) and mortality in patients (1, 2).

Despite many advantages of NIV are many when used in critically ill patients, NIV has a 40% failure rate due to patient non-compliance (3). Many studies have shown that sedation and analgesia are effective and safe for improving compliance in patients intolerant to NIV (4–7).

There are a limited number of studies on sedation protocols applied during NIV, and there is no recommended drug and no common protocol regarding sedation and analgesia in NIV (8). It has been stated that sedation, when used appropriately and with precautions, increases patient comfort and reduces the possibility of failure in patients using NIV (8, 9).

Dexmedetomidine is a potent selective α2-agonist with sedative, analgesic and anxiolytic properties (10). Many studies have shown that dexmedetomidine is useful for sedation in the ICU (11–13). In placebo-controlled studies, it has been reported that low doses of dexmedetomidine also provide sedation and analgesia, which can easily be aroused (14, 15). Remifentanil is an ultra- short -acting opioid that rapidly reaches a steady state, with an onset of action of <1 min and μ selectivity (16). Remifentanil is a safe and effective opioid that reduces NIV failure (17). Propofol is frequently used for sedation due to its short duration of action and clear awakening profile (18). Propofol is an appropriate sedative agent for NIV owing to its pharmacokinetic rate (19).

To our knowledge, no previous study has compared dexmedetomidine, remifentanil, and propofol used to provide sedation and/or analgesia, in NIV management. Our study aimed to evaluate the effects of dexmedetomidine, remifentanil, and propofol on the clinical outcomes in NIV intolerant patients.

## Materials and methods

### Patient population and design

#### Ethics statement

Ethical approval for this prospective randomized study was obtained from the Clinical Research Ethics Committee of University of Health Sciences, Yüksek Ihtisas Training and Research Hospital, Ankara, Turkey (dated 12.11.2018 and numbered 12079).

#### Patients

This prospective randomized cohort study was conducted in a tertiary ICU, between December 2018 and December 2019. Written informed consent was obtained from all patients. The patients included in the study were over 18 years of age, and had NIV intolerance, admission to the ICU, acute respiratory acidosis [partial pressure of carbon dioxide (PCO_2_) ≥ 45 mmHg], a diagnosis of COPD, respiratory rate (RR) ≥ 24 per minute, and respiratory distress, with the use of auxiliary respiratory muscles. Patients with congestive heart failure, neurologic disease, muscular disease, treatment rejection, hepatic failure, gastrointestinal bleeding, severe hypotension [mean arterial pressure (MAP) < 60 mmHg], acute cardiac ischemia, and dexmedetomidine, remifentanil, and propofol allergy were excluded from the study.

We divided patients into five groups besed on the type and dose of drugs administered dexmedetomidine low (DEX_L_), dexmedetomidine high (DEX_H_), remifentanil low (REM_L_), remifentanil high (REM_H_), and propofol (PRO). Patients underwent simple randomization using a total of 120 (23 each for DEX_L_, DEX_H_, REM_L_, REM_H_ groups, and 28 for the PRO group) closed envelopes, which declared group assignment and described the sedation protocol.

### Data collection

Gender, age (years), body mass index (BMI, kg/m^2^), ejection fraction (EF,%), Acute Physiology and Chronic Health Evaluation (APACHE) II score, comorbidities, NIV time (hours), invasive mechanical ventilation (IMV) time (days), length of intensive care unit stay (ICU LOS) (days), length of hospital stay (hospital LOS) (days), NIV complications, intubation (endotracheal intubation recordings), 30-day mortality, side effects, pH, partial pressure of carbon dioxide (PCO_2_), partial pressure of oxygen (PO_2_), Ramsay Sedation Scale (RSS) (Table 1) (20), peripheral oxygen saturation (SpO_2_),respiratory rate (RR), heart rate (HR), and mean arterial pressure (MAP) were recorded. All data were recorded at the start of the NIV, at the first, second, fourth, sixth, ninth, and twelfth hours of the NIV; and at the first hour after the end of the NIV.

**Table 1:** Ramsay sedation scale (20).

**Clinical evaluation**	**Score**
Patient is anxious and agitated or restless, or both	1
Patient is cooperative, oriented and tranquil	2
Patient responds to commands only	3
Patient exhibits brisk response to light glabellar tap or loud auditory stimulus	4
Patient exhibits a sluggish response to light glabellar tap or loud auditory stimulus	5
Patient exhibits no response	6

### Non-invasive mechanical ventilation

NIV was performed using a Servo-S ICU mechanical ventilator (Maquet Critical Care AB; Rontgenvagen, Sweden), administered intermittently through a nose-mouth mask in the pressure support ventilation (PSV) mode. The patients were ventilated with 6 cmH_2_O positive end expiratory pressure (PEEP), 12 cmH_2_O pressure support, and an inspiratory oxygen fraction (FiO_2_) of 50%. The NIV settings were meticulously adjusted during therapy based on each patient's condition after therapy began. Mechanical ventilation parameters were increased or decreased according to the patient's needs and the target saturation was at least 90%. We recorded the number of hours NIV was administered in 24 h as the “NIV time”.

In the first hour of NIV administration, NIV intolerance was assessed using the NIV intolerance score (NIS). The NIS included four points; 1, a comfortable patient tolerating NIV; 2, a mildly intolerant patient who felt some degree of discomfort and occasionally grabbed at the NIV mask; 3, moderate intolerance and discomfort (sometimes pulling), most often with NIV mask, with frequent grabbing at the mask; 4, severe NIV intolerance with an agitation unable to keep the NIV mask on the face (21). According to this scoring, patients who scored 3 and 4 were considered to have NIV intolerance.

We stopped NIV treatment in patients without acute respiratory acidosis who did not show signs of respiratory distress (such as an RR of ≥ 24 per minute and increased use of the accessory respiratory muscle), and had an SpO_2_ of 90% or more (with the inhaled oxygen flow through the oxygen mask ≤ 10 L/min). Invasive mechanical ventilation after endotracheal intubation was applied to patients who met at least two criteria; RR ≥ 45 per minute, increased amount of secretions in the trachea, acidosis with a pH value ≤ 7.25, SpO2 values ≤ 90% for at least 5 min, hemodynamic instability (HR: ≤ 60 beats/min/≥200 beats/min, MAP: ≤ 60 mmHg), impaired consciousness, and persistent/worsening respiratory failure symptoms.

### Sedatives

The first measurements were recorded when NIV treatment was initiated. A loading dose of dexmedetomidine 1 μg/kg was administered as an infusion within 10 min, after which regular infusion was started. Regular dexmedetomidine infusion was started 0.2 μg/kg/h in the DEXL group and at 0.6 μg/kg/h in the DEXH group. Any increases and/or decreases during the infusion were made at the dose rate of 0.1 μg/kg/h, according to the RSS 2–3 target. A loading dose of remifenthanyl 1 μg/kg was administered as an infusion within 30–60 s, after which regular infusion was started. Regular remifenthanyl infusion was started at 0.03 μg/kg/h in the REML group, and at 0.06 μg/kg/h in the REMH group. Any increases and/or decreases during the infusion were made at the rate of 0.025 μg/kg/h, according to the RSS 2–3 target. A loading dose of 1 mg/kg was administired as an infusion within 10 min, after which regular infusion was started. Regular propofol infusion was initiated at 0.3 mg/kg/h, and any increases and/or decreases were made at the rate of 0.1 mg/kg/h, according to the RSS 2–3 target. Hemodynamics and side effects were recorded (Table 2). Medication infusions were administered continuously for 24 h. Data were recorded at the start of NIV; at the first, second, fourth, sixth, ninth, and twelfth hours of NIV; and at the first hour after the end of the NIV.

**Table 2:** Initial dose and increasing and decreasing dose of each sedative drug.

**Drug**	**Initial dose**	**Increasing and** **decreasing** **dose**
Dexmedetomidine	0.2–0.7 μg/kg/h by continuous intravenous infusion	0.1 μg/kg/h
L (low)	0.2 μg/kg/h by continuous intravenous infusion	
H (High)	0.6 μg/kg/h by continuous intravenous infusion	
Remifentanyl	0.03–0.1 μg/kg/h by continuous intravenous infusion	0.025 μg/kg/h
L (low)	0.03 μg/kg/h by continuous intravenous infusion	
H (High)	0.06 μg/kg/h by continuous intravenous infusion	
Propofol	0.3 mg/kg/h by continuous intravenous infusion	0.1 mg/kg/h

### Statistical analysis

G^*^Power 3.1.9.4 program was used to calculate the sample size. In the priori analysis, it was planned to include at least 16 participants in each group, with medium effect size (0.3), 80% power, 5% type 1 error, and 20% type 2 error. At the end of the study, the power of the study was found to be 91% in the *post-hoc* analysis.

Histograms, q-q plots, and Shapiro-Wilk's test were used to assess data normality. The Levene's test was used to test variance homogeneity. Various tests were used to compare demographic and clinical parameters among the study groups; one-way analysis of variance (ANOVA) or Kruskal-Wallis H tests were use for continuous variables, whereas Pearson chi-square analysis or Fisher-Freeman-Halton test were used for categorical variables. Bonferroni- adjusted Dunn's test and Bonferroni- adjusted *z*- tests were performed for multiple comparison analysis. In descriptive statistics, continuous numerical variables are presented as medians [interquartile range (IQR)], and categorical variables are presented as the number of samples (%). IBM SPSS Statistics 20 (IBM Corporation, Armonk, New York, USA) was used to conduct the statistical analyses. A *p*-value of <5% was considered statistically significant.

## Results

**Figure 1:**
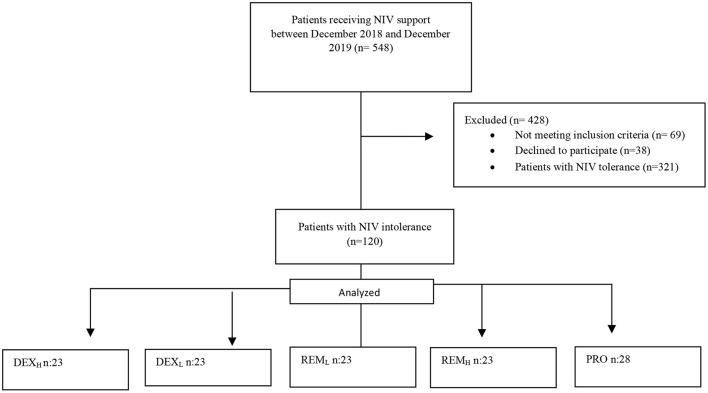
Study flow diagram. DEX_L_, Dexmedetomidine low; DEX_H_, dexmedetomidine high; REM_L_, remifentanil low; REM_H_, remifentanil high; PRO, propofol.

Between December 2018 and December 2019, 548 patients who received niv support were followed. Four hundred and twenty-eight patients were excluded from the study [Not meeting inclusion criteria (*n* = 69), declined to participate (*n* = 38), patients with NIV tolerance (*n* = 321)]. Total NIV intolerance was found to be 41.4% (*n*: 227). One hundred and twenty patients with NIV intolerance were included in the study. The DEX_L_, DEX_H_, REM_L_, and REM_H_ groups consisted of 23 patients each while the PRO group consisted of 28 patients (Figure 1). There was no difference in baseline variables other than gender distribution between the groups (*P* = 0.031). Female gender was dominant in the DEX_L_ group, while male gender was dominant in the DEX_H_, REM_L_, REM_H_, and PRO groups. The baseline characteristics of the patients were similar among the five groups. There were no differences between the groups with respect to age, BMI, EF, APACHE II score, and comorbidities (*P* = 0.993, 0.546, 0.953, 0.293, 0.783, respectively). However, diabetes mellitus (DM) differed between the groups with the highest rate observed in the PRO group. The NIV time, IMV time, ICU LOS, hospital LOS, intubation rate, and mortality were significantly different among the five groups (*P* = 0.045, 0.001, 0.001, 0.010, 0.001 and 0.041, respectively). Based on the intubation numbers, NIV failure in each group was: 2 (8.7%) in the DEX_L_ group, 3 (13%) in the DEX_H_ group, 7 (30.4%) in the REM_L_ group, 13 (56.5%) in the REM_H_ group, and 20 (71.4%) in the PRO group (*P* = 0.001). The side effects showed a significant difference among the five groups; apnea was higher in the PRO group (25%) than in the other groups (0% in the DEX_L_ group, 0% in the DEX_H_ group, 0% in the REM_L_ group, 4.3% in the REM_H_ group) (*P* = 0.001) (Table 3).

**Table 3:** Baseline characteristics of the groups and data on clinical follow-up.

	**DEX_L_**	**DEX_H_**	**DEX_H_**	**REM_H_**	**PRO**	***p*-value**
	***n*: 23**	***n*: 23**	***n*: 23**	***n*: 23**	***n*: 28**	
Gender F/M (*n*)	14/9^a^	9/14^b^	4/19^b^	6/17^b^	10/18^b^	0.031^*^
Age (year)**^#^**	66 (60–82)	74 (59–80)	71 (67–77)	72 (62–76)	71.5 (64.5–76)	0.993
BMI (kg/m^2^)**^#^**	26.4 (24.6–34.3)	26.4 (25.6–29.3)	27.5 (25–29.4)	28.4 (26.3–31.2)	30 (26.1–34.3)	0.546
EF%**^#^**	60 (57–67)	60 (58–66)	60 (55–66)	60 (58–67)	60 (55.75–65.75)	0.953
APACHE II score**^#^**	12 (10–18)	12 (8–16)	11 (10–15)	10 (8–13)	13.5 (8.25–19)	0.293
**Comorbidity** **^¤^**	10 (43.5)	12 (52.2)	10 (43.5)	13 (56.5)	16 (57.1)	0.783
DM	1 (4.3)^a^	2 (8.7)^a^	0 (0.0)^a^	0 (0.0)^a^	9 (32.1)^b^	0.001^*^
HT	1 (4.3)	3 (13)	4 (17.3)	3 (13)	4 (14.3)	0.740
CAD	1 (4.3)	3 (13)	1 (4.3)	3 (13)	1 (3.6)	0.510
AF	3 (13)	5 (21.7)	5 (21.7)	7 (30.4)	6 (21.4)	0.730
Obesity 30 ≤ BMI	8 (34.8)	5 (21.7)	5 (21.7)	6 (26)	10 (35.7)	0.691
NIV time (hour)**^#^**	12 (10–14)^a^	12 (8–16)^a^	14 (10–18)^a^	12 (9–16)^a^	15 (12–17.75)^a^	**0.045** ^*^ **^§^**
IMV time (day)**^#^**	0 (0–0)^a^	0 (0–5)^a^	0 (0–10)^ab^	0 (0–5)^ab^	3.5 (0–8.5)^b^	0.001^*^
ICU LOS (day)**^#^**	5 (4–8)^ab^	3 (2–9)^a^	6 (2–8)^abc^	10 (6–13)^bc^	9 (6.25–15.75)^c^	0.001^*^
Hospital LOS (day)**^#^**	9 (7.5–12)^a^	9 (7–16)^a^	11 (9–14.5)^a^	13 (10–19)^a^	15 (10–18.5)^a^	**0.010** ^*^ **^§^**
NIV comp**^¤^**	0 (0.0)	0 (0.0)	1 (4.3)	0 (0.0)	0 (0.0)	0.479
Intubation**^¤^**	2 (8.7)^a^	3 (13)^a^	7 (30.4)^ab^	13 (56.5)^bc^	20 (71.4)^c^	0.001^*^
Mortality**^¤^**	1 (4.3)^a^	2 (8.7)^a^	5 (21.7)^ab^	5 (21.7)^ab^	10 (35.7)^b^	0.041^*^
**Side effect** **^¤^**	1 (4.3)^a^	3 (13)^b^	1 (4.3)^a^	8 (34.8)^c^	8 (28.6)^c^	0.012^*^
Hypotension	0 (0.0)	0 (0.0)	0 (0.0)	0 (0.0)	1 (3.6)	0.507
Bradycardia	1 (4.3)	2 (8.7)	0 (0.0)	0 (0.0)	0 (0.0)	0.215
Apnea	0 (0.0)^a^	0 (0.0)^a^	0 (0.0)^a^	1 (4.3)^a^	7 (25)^b^	0.001^*^
Nausea	0 (0.0)	0 (0.0)	0 (0.0)	2 (8.7)	0 (0.0)	0.075
Thorax rigidity	0 (0.0)	0 (0.0)	0 (0.0)	2 (8.7)	0 (0.0)	0.075
Mouth dry	0 (0.0)	0 (0.0)	1 (4.3)	1 (4.3)	0 (0.0)	0.518
Hypotension + bradycardia	0 (0.0)	1 (4.3)	0 (0.0)	2 (8.7)	0 (0.0)	0.215

F, female; M, male; BMI, body mass index (kg/m^2^); EF, ejection fraction (%); APACHE II score, Acute Physiology and Chronic Health Evaluation II score; CAD, coronary artery disease; COPD, chronic obstructive pulmonary disease; DM, diabetes mellitus; HT, hypertension; AF, atrial fibrillation; NIV time, noninvaziv ventilasyon time (hours); IMV time, invasive mechanical ventilation time (days); ICU LOS, length of intensive care unit stay (days); Hospital LOS length of hospital stay (days);NIV comp, noninvaziv ventilasyon complication; IQR, interquartile range; SD, standard deviation (

* *P* < 0.05,

§ *P* > 0.005).

Different superscripts among groups indicate a statistically significant difference between groups. Significant results are shown in bold.

¤ Results are expressed as *n* (%).

# Results are expressed as median (IQR).

During continuous intravenous infusion in all groups except the PRO group, the pH level gradually good compared to the baseline values. There were significant differences between the groups at the sixth, ninth, twelfth, and first hour after NIV. There was a statistically significant difference between the REM_H_/DEX_H_ groups at the sixth hour (*P* = 0.015), and between the REM_H_/DEX_H_ groups and the PRO/DEX_H_ groups at both the ninth (*P* = 0.02, 0.012, respectively) and twelfth (*P* = 0.004 and 0.028, respectively) hours. There was a difference between the PRO/DEX_L_ groups, PRO/DEX_H_ groups, REM_L_/DEX_L_ groups, and REM_L_/DEX_H_ groups at the first hour after NIV (*P* = 0.001, 0.001, 0.04, 0.039, respectively).

**Figure 2:**
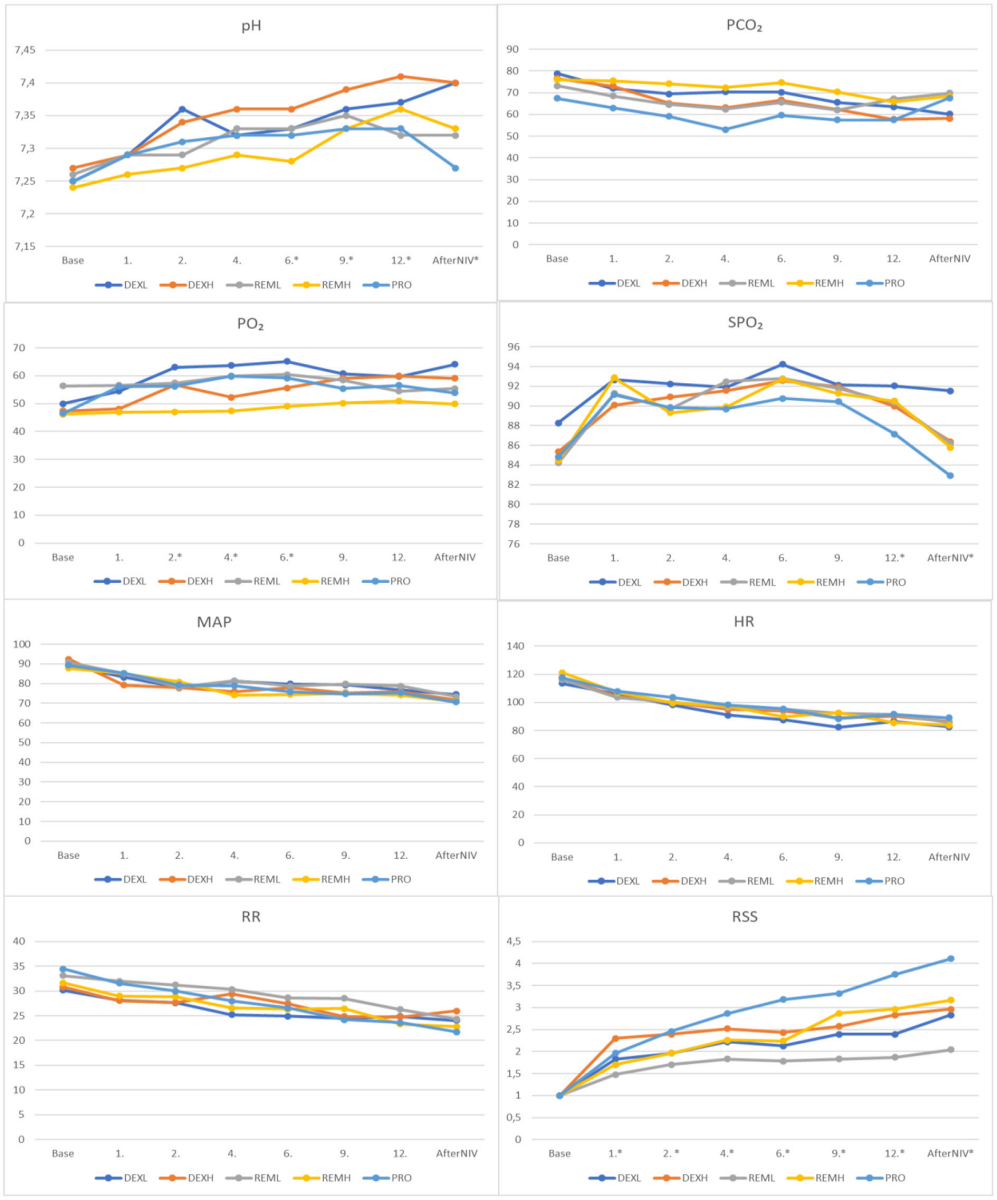
Comparison of study groups according to blood gas results, RSS and monitoring records. RSS, Ramsay Sedation Scale; SpO_2_, peripheral oxygen saturation; RR, respiratory rate; HR, heart rate; MAP, mean arterial pressure; pH, potential of hydrogen; PCO_2_, partial pressure of carbon dioxide; PO_2_, partial pressure of oxygen. ^*^P < 0.05.

There were significant differences in the PaO_2_ between the groups at the second (REM_H_/DEX_L_), fourth (REM_L_/REM_H_), and sixth (REM_H_/DEX_L_) hours (*P* < 0.05). Significant differences were also found in the SpO_2_ between the groups at the twelfth hour (PRO/DEX_L_), and the first hour after NIV (PRO/DEX_L_) (*P* < 0.05). There were no significant differences in the PaCO_2_, HR, RR, and MAP between the groups. The Ramsay Sedation Scale (RSS) differed significantly between the groups at all times other than baseline values (*P* < 0.05). The highest RSS values were recorded in the PRO group at the second, fourth, sixth, ninth, and twelfth hours, and the first hour after NIV. The lowest values were observed in the REM_L_ group at all times except the baseline. Except for the REML group, the target sedation was reached at the second hour in the other groups (Figure 2).

## Discussion

We frequently use NIV therapy in patients hospitalized in the ICU due to type 2 respiratory failure, as is the trend worldwide. Although NIV has many advantages when used in critically ill patients, a 40% failure rate is observed due to patient non-compliance. The lack of tolerance to NIV makes its application difficult (3). In this study, we included 120 patients with NIV intolerance in tertiary ICU and evaluated three drugs (dexmedetomidine, remifentanil, and propofol) used for sedation and/or analgesia, based on their clinical results in five groups. In the groups that were given dexmedetomidine (DEX_L_, and DEX_H_), NIV failure, mortality, ICU LOS, and hospital LOS were lower than in the other groups.

The “ISCCM (Indian Society of Critical Care Medicine) Guidelines” were published in 2020, but did not recommend any drug specifically. They suggested that sedation in patients undergoing NIV can be used in an ICU setting, with very close monitoring, and paying attention to the signs of NIV failure (8). It has been stated that sedation, when used appropriately and with precautions, reduces the possibility of failure in patients and increases patient comfort using NIV (8, 9).

Agitation during NIV may be caused by various factors such as fear, pain, fever, anxiety, sleep deprivation, and hypoxia (22). The sedation applied during NIV facilitates and calms ventilation and improves patient compliance. It also regulates autonomic system responses to stress such as hypertension and tachycardia and can also reduce the rate of NIV failure (5, 23, 24). In this prospective study, when we examined the groups based on the number of intubations, NIV failure in the dexmedetomidine groups was low compared to that in the other groups. Many studies have shown that sedation provided by dexmedetomidine, midazolam, propofol, and remifentanil during NIV is effective and safe (8, 25). We consider that the safest drug is dexmedetomidine, since NIV failure was lowest in patients receiving this drug.

Dexmedetomidine provides sedo-analgesia without causing respiratory depression (10). It does not cause respiratory depression even when deep sedation levels are achieved (26). Consistent with these studies, we also did not observe apnea at low or high doses of dexmedetomidine.

Propofol negatively affects the respiratory drive and gas Exchange, in proportion to the infusion rate of the sedation dose (19). Clinicians use drugs, which may impair the respiratory and cough reflexes, carefully (27). We did not study propofol at high doses due to the high possibility of this side effect. Despite its risk, it has been shown that propofol can be used effectively with target-controlled infusion (28). In our study, apnea developed in 25% patients in propofol.

It is well-known that the use of opioids for sedation causes respiratory depression (29, 30). However, it has been reported that remifentanyl infusion can be administered safely at doses of 0.05–0.1 μg/kg/min in patients with spontaneous ventilation (31). However, Cavaliere et al. concluded that remifentanyl infusion at a dose higher than 0.05 μg/kg/min may inhibit the respiratory impulse (30). In our study, apnea was observed in only one (4.3%) patient in the REM_H_ group (0.06 μg/kg/min), while no case of apnea was observed in the REM_L_ group (0.03 μg/kg/min).

Bradycardia may occur when remifentanil is administered rapidly and in high doses. Low doses of remifentanil do not cause significant changes in blood pressure (32). In this study, coexistence of hypotension and bradycardia (HR: <60 beats/min, MAP: < 60 mmHg) was recorded in two (8.7%) patients in the REM_H_ group. Similar to the studies showing that dexmedetomidine is associated with a high incidence of bradycardia and hypotension (33, 34), we also found a higher incidence of bradycardia in the DEX_L_ (4.3%) and DEX_H_ (8.7%) groups than in the other groups (0%), but this difference was not statistically significant (*P* = 0.215).

Opioids, are frequently added to the treatment regimens in the ICU for cardiovascular diseases, because of their protective effect on the heart tissue (35). Remifentanil is an ultra- short -acting opioid that rapidly reaches a steady state, with an onset of action of <1 min and μ selectivity (34). The elimination half life of remifentanil is <10 min, independent of kidney function, liver function, and infusion time (36). Remifentanil is a safe and effective opioid that reduces NIV failure (17). According to a recent study, there was no significant difference between dexmedetomidine and remifentanil in terms of NIV failure and other clinical outcomes (tracheostomy, length of ICU stay, length of hospital stay, and in hospital mortality). The side effects of both drugs were rare (chest wall rigidity in one patient with remifentanil, and severe hemodynamic instability requiring intubation with dexmedetomidine). In addition, NIV failure was avoided in more than 80% of the patients enrolled in this study (21). We also did not find any differences between the groups in terms of the incidence of side effects (*P* > 0.05). However, we observed better clinical results in the DEX_L_ and DEX_H_ groups. IMV time, ICU LOS, hospital LOS were significantly reduced in these groups compared with the other groups (*P* < 0.05). Mortality and NIV failure were also lower in these groups compared to the other groups (*P* < 0.05).

In summary, NIV has become increasingly important in the treatment of both hypercapnic and hypoxemic acute respiratory failure. NIV reduces the need for IMV. NIV failure defined as the need for endotracheal intubation, is one of the biggest problems in NIV patients. Patient rejection and discomfort are among the reasons for failure. Therefore, patient comfort must be monitored. Non-pharmacological methods and analgo-sedative drug schemes are used to manage agitation during NIV. In the case of agitation, the addition of sedatives to therapy should be considered. There is evidence that sedation reduces the NIV failure rate. In the selection of the drug, clinical and side effects should be considered. Sedative drugs should be administered in ICU, in the presence of well-trained personnel in airway emergency management, with monitoring of vital signs and depth of sedation (37).

One limitation of this study was that it was conducted in a single center and with a limited number of participants. In this study, in which we evaluated the sedative effects of the drugs used with the target RSS 2–3, we did not add analgesic drugs in addition to propofol, which has no analgesic effect. Using drugs in different doses, we aimed to establish the best safe and effective evidence-based dosing recommendation for sedatives used in NIV intolerance. We did not study propofol at high doses due to the high possibility of side effect.

In conclusion, in this prospective study, we compared the results of three drugs (propofol, dexmedetomidine, and remifentanil) in patients with NIV intolerance. Seventy-five patients (62.5%) in total become tolerant of NIV after starting the drugs. Sedation used in patients with NIV intolerance increased the success of NIV. NIV failure, mortality, ICU LOS, IMV time, and hospital LOS were found to be lower with dexmedetomidine. With the use of low doses, the incidence of side effects decreased, the target sedation level was reached, and NIV intolerance decreased.

We believe that this study, supported by multicenter studies with larger sample sizes in the future, will help improve outcomes, in patients with NIV intolerance, who are hospitalized in the ICU due to respiratory failure.

## Data availability statement

The original contributions presented in the study are included in the article/supplementary material, further inquiries can be directed to the corresponding author.

## Ethics statement

Written informed consent was obtained from the individual(s), and minor(s)' legal guardian/next of kin, for the publication of any potentially identifiable images or data included in this article.

## Author contributions

All authors listed have made a substantial, direct, and intellectual contribution to the work and approved it for publication.

## Conflict of interest

The authors declare that the research was conducted in the absence of any commercial or financial relationships that could be construed as a potential conflict of interest.

## Publisher's note

All claims expressed in this article are solely those of the authors and do not necessarily represent those of their affiliated organizations, or those of the publisher, the editors and the reviewers. Any product that may be evaluated in this article, or claim that may be made by its manufacturer, is not guaranteed or endorsed by the publisher.
